# SPLUNC1 regulation in airway epithelial cells: role of toll-like receptor 2 signaling

**DOI:** 10.1186/1465-9921-11-155

**Published:** 2010-11-05

**Authors:** Hong Wei Chu, Fabienne Gally, Jyoti Thaikoottathil, Yvonne M Janssen-Heininger, Qun Wu, Gongyi Zhang, Nichole Reisdorph, Stephanie Case, Maisha Minor, Sean Smith, Di Jiang, Nicole Michels, Glenn Simon, Richard J Martin

**Affiliations:** 1 Department of Medicine, National Jewish Health, and the University of Colorado Denver, Denver, CO, USA; 2 Department of Immunology, National Jewish Health, and the University of Colorado Denver, Denver, CO, USA; 3 Department of Pathology, University of Vermont, Burlington, VT, USA

## Abstract

**Background:**

Respiratory infections including *Mycoplasma pneumoniae *(Mp) contribute to various chronic lung diseases. We have shown that mouse short palate, lung, and nasal epithelium clone 1 (SPLUNC1) protein was able to inhibit Mp growth. Further, airway epithelial cells increased SPLUNC1 expression upon Mp infection. However, the mechanisms underlying SPLUNC1 regulation remain unknown. In the current study, we investigated if SPLUNC1 production following Mp infection is regulated through Toll-like receptor 2 (TLR2) signaling.

**Methods:**

Airway epithelial cell cultures were utilized to reveal the contribution of TLR2 signaling including NF-κB to SPLUNC1 production upon bacterial infection and TLR2 agonist stimulation.

**Results:**

Mp and TLR2 agonist Pam3CSK4 increased SPLUNC1 expression in tracheal epithelial cells from wild type, but not TLR2^-/- ^BALB/c mice. RNA interference (short-hairpin RNA) of TLR2 in normal human bronchial epithelial cells under air-liquid interface cultures significantly reduced SPLUNC1 levels in Mp-infected or Pam3CSK4-treated cells. Inhibition and activation of NF-κB pathway decreased and increased SPLUNC1 production in airway epithelial cells, respectively.

**Conclusions:**

Our data for the first time suggest that airway epithelial TLR2 signaling is pivotal in mycoplasma-induced SPLUNC1 production, thus improving our understanding of the aberrant SPLUNC1 expression in airways of patients suffering from chronic lung diseases with bacterial infections.

## Background

Palate, lung, and nasal epithelium clone (PLUNC) are a recently described family of proteins that have been predicted to exert host defense functions [1-4]. Among the 10 PLUNC proteins described so far, short PLUNC1 (SPLUNC1) has been localized to large airway epithelium in humans and mice [1,5,6]. Our recent publication suggests that recombinant mouse SPLUNC1 protein inhibits the growth of *Mycoplasma pneumoniae *(Mp), an atypical bacterium contributing to several common respiratory diseases including community-acquired pneumonia and asthma [7,8]. In line with our findings, a recent study by Zhou and colleagues further revealed that human SPLUNC1 protein impaired the growth of Gram-negative bacterium *Pseudomonas aeruginosa*, a major cause of infection in chronic lung diseases such as cystic fibrosis [9]. However, Bartlett et al later did not show an antimicrobial effect of recombinant human SPLUNC1 protein on *Pseudomonas aeruginosa *[10]. Such discrepancy emphasizes the need to further characterize the function of SPLUNC1.

To date, SPLUNC1 regulation under physiological or pathological conditions remains poorly understood [11-14]. We and other investigators have clearly demonstrated the down-regulation of SPLUNC1 in human bronchial and nasal epithelial cells by the Th2 cytokine IL-13 [7,15]. On the other hand, Bingle and co-workers found increased SPLUNC1 protein in airway epithelium of patients with cystic fibrosis [16]. It remains unclear if bacteria (e.g., Mp) or their products directly modulate SPLUNC1 production in airway epithelial cells. If so, what are the mechanisms underlying SPLUNC1 regulation? Studies from our group and others have suggested that signaling through Toll-like receptors (TLRs) is critical to host defense against various pathogens including Mp [17-23]. Specifically, we demonstrated that Mp infection in airway epithelial cells and mouse lungs activated TLR2 signaling (e.g., increased TLR2 expression and NF-κB activation). Mp infection in TLR2 deficient mice failed to induce inflammatory cytokine (e.g., IL-6) and airway epithelial mucin production [17]. In the current study, we hypothesize that SPLUNC1 expression is in large part under the control of TLR2 signaling (e.g., dependence on TLR2 expression and NF-κB activation). To test our hypothesis, we utilized human and mouse airway epithelial cell cultures to determine if TLR2 signaling is involved in SPLUNC1 expression following Mp infection or TLR2 agonist stimulation. We found that airway epithelial TLR2 signaling is pivotal in SPLUNC1 production.

## Methods

### Culture of NCI-H292 cells

A human pulmonary mucoepidermoid carcinoma cell line NCI-H292 (ATCC, Manassas, VA) was utilized to perform a time course and a dose response of SPLUNC1 production following Mp infection or TLR2 agonist stimulation. NCI-H292 cells at 90-100% confluence were infected with Mp at 1, 5 and 10 colony forming unit [cfu]/cell, or stimulated with a TLR2 agonist Pam(3)-Cys-Ser-Lys-Lys-Lys-Lys-OH (Pam3CSK4) at 10, 100 and 1000 ng/ml for up to 48 hrs. The supernatants were collected for SPLUNC1 protein measurement using an ELISA. Cells were lyzed in Trizol reagent (Gibco BRL, Rockville, MD) for RNA extraction to perform quantitative real-time PCR of SPLUNC1 mRNA, or processed in cell lysis buffer to carry out Western blot analysis of SPLUNC1 protein.

### Air-liquid interface (ALI) culture of mouse tracheal epithelial cells

ALI cultures of mouse tracheal epithelial cells were carried out as previously reported by our group [24-26] to study the direct role of TLR2 and NF-κB in SPLUNC1 regulation. All experimental animals used in this study were covered by a protocol approved by our Institutional Animal Care and Use Committee. Briefly, tracheas from mice were isolated and digested with 0.1% protease, and the released cells from tracheas were plated (about 4 × 10^4 ^cells/cm^2^) on collagen-coated polyester Transwell inserts of 12 mm in diameter (pore size, 0.4 μm; Corning Inc., Corning, NY, USA). After 7 days of immersed culture, tracheal epithelial cells reached 100% confluence and were shifted to an ALI condition by removing all but 50 μl of the apical medium. Cells under the ALI condition are known to undergo mucociliary cell differentiation, thus mimicking in vivo airway epithelial cell biology. On day 10 of ALI culture, epithelial cells were treated with Mp (10 cfu/cell, strain FH, ATCC 15531, ATCC, Manassas, VA) at the apical side, Pam3CSK4 (1 μg/ml, InvivoGen, San Diego, CA) at both apical and basolateral sides, or medium alone as a control. After 48 hrs of treatments, 200 μl of PBS was applied to the apical surface of ALI cell culture and incubated for 5 minutes at room temperature to obtain the apical supernatants for SPLUNC1 protein measurement using Western blot analysis.

### TLR2 RNA interference in normal human bronchial epithelial cells (NHBE)

A VSV-G pseudotyping approach was utilized to transduce human TLR2 short hairpin (sh)RNA encoded in a lentiviral vector (pLL3.7) to primary normal human bronchial epithelial cells. The oligonucleotide sequences that encode shRNA of hTLR2 are: Sense - 5'- TGCAGCTCA-GGATCTTTAAATTCAAGAGATTTAAAGATCCTGAGCTGCTTTTTTC-3'; Anti-sense - 5'-TCGAGAAAAAAGCAGCTCAGGATCTTTAAATCTCTTGAATTTAAAGATCCTGAGCT-GCA-3'. NHBE were isolated from bronchial tissues of three donors without any lung diseases or smoking history through the International Institute for the Advancement of Medicine (IIAM) (Jessup, PA). Cells at passage one were used for lentiviral transduction experiments.

Antiparallel pairs of human TLR2 oligonucleotides were ordered from the IDT laboratories and TLR2 shRNA encoded in pLL3.7 was generated as previously described [7,27]. Briefly, epithelial cells were cultured in 6-well culture plates (2 × 10^5 ^cells/well) under the immersed condition until about 60% confluence when they were transduced with either pLL3.7-shTLR2 (50 focus-forming units [ffu]/cell) or pLL3.7-sh firefly luciferase (an irrelevant gene control, 50 ffu/cell) once daily for three consecutive days. Forty-eight hrs after the last transduction, cells from each condition were collected to verify TLR2 gene knockdown. The remaining cells were used for ALI culture.

### ALI culture of NHBE

NHBE that were transduced with either pLL3.7-shTLR2 or pLL3.7-shFirefly luciferase were cultured under ALI conditions to determine if gene knockdown of TLR2 affects SPLUNC1 production following Mp infection or a TLR2 agonist stimulation. ALI culture was performed by plating the lentivirus-transduced epithelial cells onto collagen-coated 12-well transwell plates at 4 × 10^4 ^cells/cm^2 ^as previously reported [24]. On day 10 of ALI culture, cells were treated with Mp (10 cfu/cell), Pam3CSK4 (1 μg/ml) or cell culture medium (control). At 48 hr post treatments, apical supernatants were collected as described for mouse tracheal epithelial cells to measure SPLUNC1 protein levels using an ELISA.

### Effects of an NF-κB inhibitor on Mp-induced SPLUNC1 production

To determine the role of NF-κB in SPLUNC1 production, we utilized an NF-B inhibitor helenalin (Calbiochem, San Diego, CA) in NHBE. Briefly, NHBE at day 10 of ALI culture were pre-treated with helenalin (10 μM in 0.1% DMSO) or 0.1% DMSO (negative control) for 2 hrs, followed by Mp (10 cfu/cell) infection or cell culture medium (control) for 48 hrs. Apical supernatants were collected for SPLUNC1 protein measurement using an ELISA. The cells were processed for nuclear protein extraction using a Nuclear Extract Kit (Active Motif, Carlsbad, CA) per manufacturer's instruction, followed by an ELISA-based assay (Active Motif) to quantify nuclear NF-B p65 activity levels.

### Direct ELISA for human SPLUNC1 protein detection

A direct SPLUNC1 ELISA was utilized to measure human SPLUNC1 protein as previously reported by our group [7]. Briefly, recombinant human SPLUNC1 protein and supernatants of cultured human airway epithelial cells were coated onto a 96-well Immulon 2HB plate (Fisher Scientific, Pittsburgh, PA, USA), followed by incubations with a mouse anti-SPLUNC1 antibody (1 μg/ml, R&D Systems), biotinylated anti-mouse antibody and avidin-biotin peroxidase complex. The plate was developed using a peroxidase substrate (TMB) and read using a plate reader.

### Western blot analysis of mouse and human SPLUNC1 protein

As no ELISA is available to detect SPLUNC1 protein in mouse samples, we utilized Western blot to quantify SPLUNC1 protein. Briefly, 15 μl of cell culture supernatant was electrophoresed on 10% SDS-PAGE, transferred onto nitrocellulose membrane, blocked with the Western blocking buffer, and then incubated with a goat anti-mouse SPLUNC1 antibody (R&D Systems) overnight at 4°C. After washes in PBS with 0.1% Tween-20, the membranes were incubated with an anti-IgG conjugated with a fluorescent dye (e.g., IRDye^® ^800), and detected by using the Odyssey Imaging System. Densitometry was then performed to quantify SPLUNC1 protein levels. Intracellular SPLUNC1 protein of human epithelial cells was similarly examined as the mouse counterpart using the Western blot analysis.

### Quantitative real-time RT-PCR

Levels of SPLUNC1 mRNA in epithelial cells were determined by reverse transcription (RT), followed by real-time quantitative PCR. Total RNA was extracted using TRIzol reagent (Gibco BRL, Rockville, MD). RT was performed using 1 μg of total RNA and random hexamers in a 50 μl reaction (Applied Biosystems, Foster City, CA). Primers and probe for human (Genbank accession #: NM_016583) SPLUNC1 genes were designed using Primer Express software (Applied Biosystems). Human SPLUNC1: forward primer, 5'-GGGCCTGTTGGGCATTCT-3'; reverse primer, 5'-CCTCCTCCAGGCTTCAGGAT-3'; probe, 5'-AAACCTTCCGCTCCTGGA- 3'. PCR was performed on the ABI Prism 7700 sequence detection system. The 25 μl PCR reaction contained 30 ng cDNA, 100 nM fluoregenic probe and 200 nM primers and other components from the TaqMan RT-PCR kit. Housekeeping gene GAPDH was also evaluated. The comparative threshold cycle (C_T_) method was employed to determine the relative gene expression levels by using one of the control conditions as the baseline level (i.e., 1) [28].

### Production of recombinant human SPLUNC1 protein and antimicrobial assay

Recombinant human SPLUNC1 protein was generated using the baculovirus expression system. Based on the cDNA sequence of human SPLUNC1 (Genbank accession #: NM_016583), PCR was performed using primers containing restriction enzyme sites (*EcoRI *and *Nhe*I) to generate fragments covering the entire SPLUNC1 protein. With verification of DNA sequence, the SPLUNC1 cDNA was then subcloned into a transfer vector (a modified p479 vector with histidine cDNA at the C-terminus) [29] to generate the recombinant SPLUNC1 baculovirus in SF9 cells to infect High-Five (Invitrogen) insect cells to produce recombinant protein, which was collected from the culture media, purified by using a His-column (Promega), followed by loading the protein onto an ion exchange column (MonoQ column) for further purification (usually > 99% pure). The purity and specificity of recombinant protein was confirmed by using SDS-PAGE, Western blot and mass spectrometry.

We tested the antimicrobial activity of SPLUNC1 protein by incubating Mp (4 × 10^4 ^cfu/ml) with recombinant human SPLUNC1 protein (1-10 μg/ml) in 96-well tissue culture plates (100 μl SP-4 broth/well) for 2 hrs, a typical time for bactericidal assay in Gram-negative bacteria. Mp in the supernatants was then plated on pleuropneumonia-like organism (PPLO) agar plates and incubated at 37°C, 5% CO2 for a week to quantify Mp. The above SPLUNC1 protein dose range selection was based on our SPLUNC1 protein measurements in airway epithelial lining fluid of normal human subjects (n = 12) who had no history of respiratory diseases or cigarette smoking, and had normal pulmonary function (e.g., FEV1 > 80%). The age (years) of normal subjects (7 males and 5 females) was 33.1 ± 3.0. SPLUNC1 protein in bronchoalveolar lavage fluid of normal human subjects was measured using a direct SPLUNC1 ELISA. After normalization of dilution factor using serum urea/BAL urea ratio, SPLUNC1 protein concentration in airway epithelial lining fluid. was calculated at 5.3 ± 2.1 μg/ml.

### Statistical analysis

For normally distributed data, one-way analysis of variance (ANOVA) was used for multiple comparisons, and a Tukey's post hoc test was applied where appropriate. Student's *t *test was used when only two groups were compared. Non-normally distributed data were compared using the Wilcoxon rank-sum test. A p value ≤ 0.05 was considered significant.

## Results

### Mp or a TLR2 agonist increases SPLUNC1 expression in NCI-H292 cells

**Figure 1:**
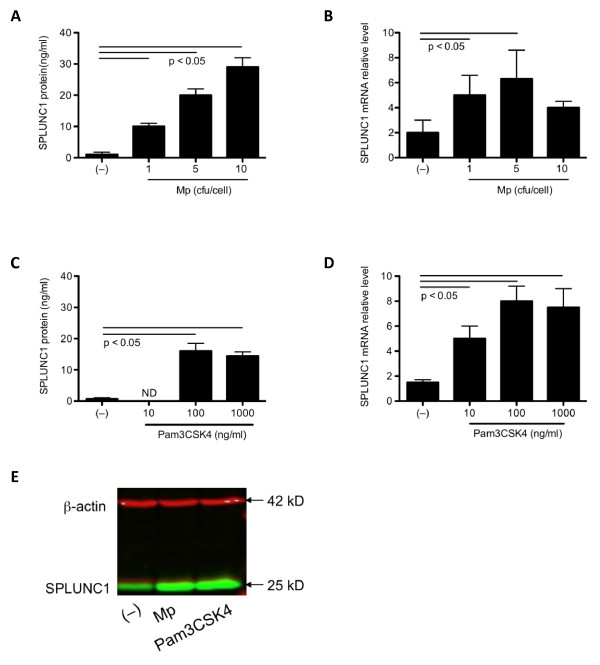
**A dose response of SPLUNC1 production in NCI-H292 cells**. Cells were cultured in 6-well plates with *Mycoplasma pneumoniae *(Mp, 1 - 10 cfu/cell) or a TLR2 agonist (Pam3CSK4, 10 - 1000 ng/ml) for 48 hrs, followed by collection of supernatants and cell lysates for SPLUNC1 mRNA and protein measurements, respectively. Data are expressed as means ± SEM (N = 6 replicates). **(A) **Mp increased SPLUNC1 protein levels in cell supernatants in a dose-dependent manner. **(B) **Mp increased SPLUNC1 mRNA expression at 1 and 5 cfu/cell. **(C) **Pam3CSK4 at 100 and 1000 ng/ml significantly augmented SPLUNC1 protein levels in cell supernatants. ND = Not detectable. **(D) **Pam3CSK4 enhanced SPLUNC1 mRNA expression in a dose-dependent fashion. **(E) **Western blot analysis of intracellular SPLUNC1 protein and β-actin (a protein loading control) in Mp (10 cfu/cell) and Pam3CSK4 (100 ng/ml)-treated cells using the Odyssey Imaging System (two color detection). Both Mp and Pam3CSK4 increased SPLUNC1 protein.

We utilized NCI-H292 cells to study the time course and dose response of SPLUNC1 expression following Mp infection. Cells were infected with Mp at 1, 5 and 10 cfu/cell for 24 and 48 hrs. Mp-infected cells, as compared to non-infected cells, demonstrated a significant increase of SPLUNC1 mRNA (up to 3-fold) and protein in a dose-dependent manner at 48 hr post infection (Figure 1A &1B). At 24 hr, Mp did not significantly increase SPLUNC1 expression (data not shown).

We also determined whether a TLR2 agonist was able to up-regulate SPLUNC1 expression in NCI-H292 cells. After 48 hrs, Pam3CSK4 at 100 and 1000 ng/ml, but not at 10 ng/ml, significantly increased SPLUNC1 protein levels in cell supernatants (Figure 1C). Pam3CSK4 also increased SPLUNC1 mRNA in a dose-dependent manner up to a dose at 100 ng/ml (Figure 1D). At 1000 ng/ml, Pam3CSK4 did not further increase SPLUNC1 mRNA expression.

Intracellular SPLUNC1 protein was also increased (about 2-fold) in Mp-infected or Pam3CSK4-stimulated cells (Figure 1E).

### TLR2^-/- ^mouse tracheal epithelial cells fail to increase SPLUNC1 upon Mp infection or TLR2 agonist stimulation

**Figure 2:**
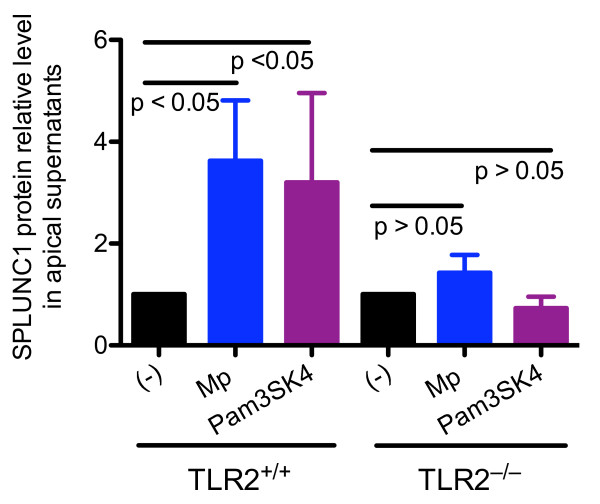
**Effects of *Mycoplasma pneumoniae *(Mp) and a TLR2 agonist (Pam3CSK4) on SPLUNC1 production in cultured mouse tracheal epithelial cells**. Tracheal epithelial cells from TLR2^+/+ ^and TLR2^-/- ^mice on the BALB/c background were isolated and cultured under the air-liquid interface (ALI) conditions for 10 days as described in the Methods section. After 48 hrs of Mp (10 cfu/cell) or Pam3CSK4 (1 μg/ml) treatment, SPLUNC1 protein levels in apical supernatants of tracheal epithelial cells were measured using Western blot, quantified using densitometry, and normalized to non-treated (-) cells to obtain SPLUNC1 protein relative levels. As compared to the non-treatment control (-), Mp or Pam3CSK4 treatment in tracheal epithelial cells from TLR2^+/+^, but not TLR2^-/- ^mice, significantly increased SPLUNC1 protein levels. Data are expressed as means ± SEM (N = 3-4 replicates).

To demonstrate the contribution of TLR2 to airway epithelial SPLUNC1 production following Mp infection, TLR2^-/- ^and TLR2^+/+ ^BALB/c mouse tracheal epithelial cells under the ALI conditions were treated with Mp or Pam3CSK4. After 48 hrs of Mp infection or Pam3CSK4 stimulation, TLR2^+/+ ^tracheal epithelial cells significantly increased SPLUNC1 protein levels in the apical supernatants. However, Mp infection or Pam3CSK4 treatment in TLR2^-/- ^tracheal epithelial cells minimally affected SPLUNC1 protein production (Figure 2). These results suggest that TLR2 stimulation can directly induce SPLUNC1 production, and an intact TLR2 signaling is necessary for SPLUNC1 induction following Mp infection.

### SPLUNC1 regulation in NHBE

**Figure 3:**
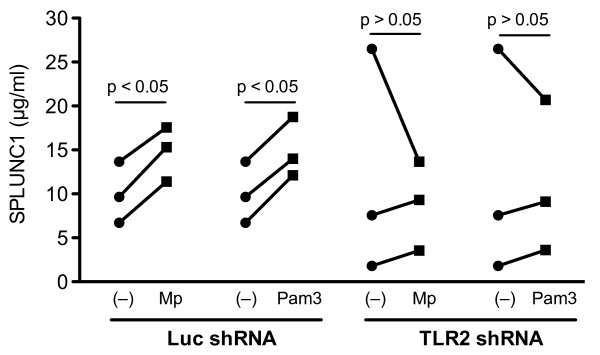
**TLR2 dependence of SPLUNC1 production in normal human bronchial epithelial cells (NHBE)**. NHBE (N = 3) were transduced with firefly luciferase short hairpin RNA (Luc shRNA, control) or TLR2 short hairpin RNA (TLR2 shRNA). Thereafter, cells were seeded onto 12-well transwell plates for air-liquid interface (ALI) culture for 10 days, and were then treated with or without *Mycoplasma pneumoniae *(Mp, 10 cfu/cell) or Pam3CSK4 (1 μg/ml) for 48 hrs. Apical supernatants were collected for SPLUNC1 protein measurement using an ELISA. The paired *t *test (for normally distributed data under Luc shRNA conditions) or Wilcoxon matched pairs test (for non-parametric data under TLR2 shRNA conditions) was used to analyze the treatment effect of Mp or Pam3CSK4 (Pam3) on SPLUNC1 protein levels. While Mp and Pam3CSK4 increased SPLUNC1 in NHBE transduced with Luc shRNA, they failed to do so in TLR2 shRNA-transduced cells.

Having shown TLR2 involvement in SPLUNC1 induction upon Mp infection or Pam3CSK4 stimulation in primary mouse tracheal epithelial cells, we tested its role in SPLUNC1 production in NHBE by knocking-down TLR2 expression. As shown in Figure 3, Mp infection or Pam3CSK4 stimulation significantly increased SPLUNC1 protein in apical supernatants of well-differentiated NHBE transduced with firefly luciferase shRNA (an irrelevant gene control). In contrast, TLR2 shRNA-transduced NHBE did not demonstrate an increase of SPLUNC1 protein after Mp or Pam3CSK4 treatment. Real-time PCR analysis demonstrated that TLR2 shRNA transduction resulted in a 5.2-fold reduction (5.2 ± 0.3 vs. 1, p < 0.05) of TLR2 expression as compared to the control (luciferase shRNA transduction).

### Role of NF-κB in SPLUNC1 regulation

Since Mp infection has been shown to increase TLR2 expression and to activate NF-κB [17], we determined if NF-κB may contribute to SPLUNC1 up-regulation following Mp infection.

**Figure 4:**
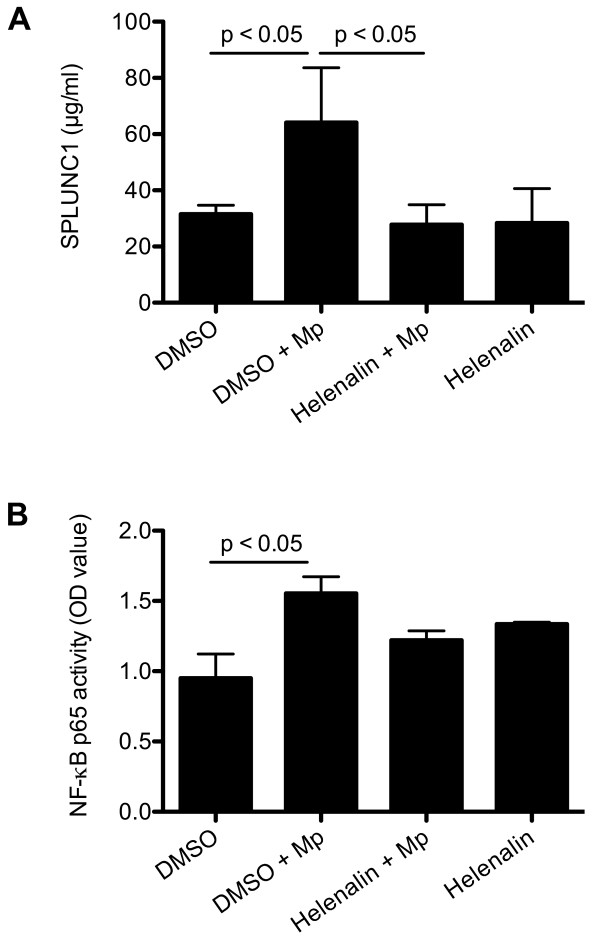
**Effects NF-**κ**B on SPLUNC1 production in normal human bronchial epithelial cells (NHBE)**. NHBE were cultured under air-liquid interface (ALI) conditions. At day 10 of ALI culture, cells were treated with an NF-κB p65 inhibitor helenalin (10 μM) for 2 hrs, followed by *Mycoplasma pneumoniae *(Mp, 10 cfu/cell) infection for 48 hrs. (A) Helenalin inhibited Mp-induced SPLUNC1 protein at the apical surface of NHBE; (B) NF-κB p65 activity was measured in the extracted nuclear proteins of NHBE using an ELISA-based assay (Active Motif, Carlsbad, CA). Mp significantly increased NF-κB p65 activity, which tended (p = 0.07) to be decreased by helenalin. Data are expressed as means ± SEM (n = 3 replicates).

We first utilized an NF-κB inhibitor helenalin in NHBE under the ALI conditions. Helenalin is an anti-inflammatory sesquiterpene lactone from Arnica, and has been shown to selectively alkylate the p65 subunit of NF-κB [30]. In the absence of Mp infection, helenalin alone did not significantly affect SPLUNC1 or NF-κB activity. However, after 24 hrs of Mp infection, helenalin significantly reduced Mp-induced SPLUNC1 production (Figure 4A), and tended (p = 0.07) to reduce NF-κB p65 activity (Figure 4B).

**Figure 5:**
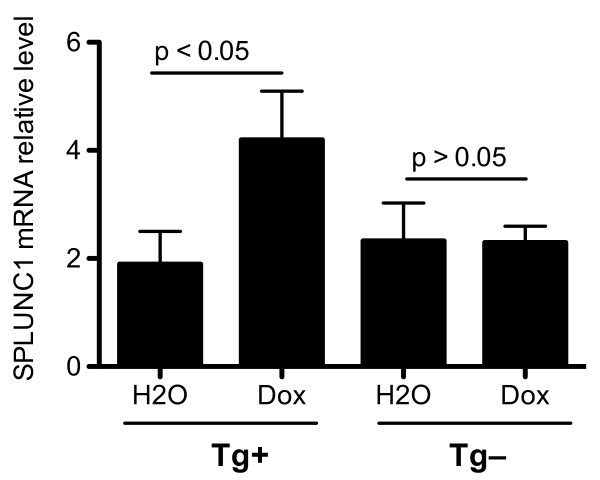
**Effects NF-**κ**B pathway on SPLUNC1 production in primary mouse tracheal epithelial cells**. Tracheal epithelial cells from CC10-tetracycline-inducible CA-IKKβ (CC10-CA-IKKβ) transgene positive and negative C57BL/6 mice were isolated and cultured under the air-liquid interface conditions for 10 days. Cells were then treated with water (H_2_O) or doxycycline (Dox) for 48 hrs. SPLUNC1 mRNA in epithelial cells was quantified by using real-time PCR. As compared to H_2_O treatment, Dox significantly increased SPLUNC1 mRNA levels in epithelial cells from CC10-CA-IKKβ transgene positive (Tg+), but not from transgene negative (Tg-) mice. Data are expressed as means ± SEM (N = 4 replicates).

To demonstrate a direct role of NF-κB pathway in SPLUNC1 production, we performed ALI cultures of tracheal epithelial cells from transgenic mice expressing a doxycycline (Dox)-inducible constitutively active (CA) version of inhibitor of κB (IκB) kinase-beta (IKKβ) under transcriptional control of the rat CC10 promoter (CC10-CA-IKKβ). Previous studies have shown selective airway epithelial NF-κB activation after Dox administration [31]. As shown in Figure 5, Dox treatment for 48 hrs, as compared with the control (H_2_O), significantly increased SPLUNC1 levels in cells from CC10-CA-IKKβ transgene positive mice, but not in those from the transgene negative mice.

### Recombinant human SPLUNC1 protein inhibits Mp growth

**Figure 6:**
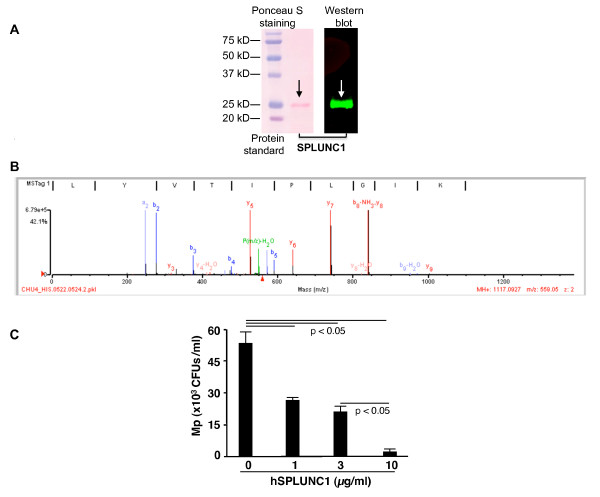
**Purity, specificity and antimicrobial activity of recombinant human SPLUNC1 protein**. **(A) **Left-panel - Two μg of SPLUNC1 protein was electrophoresed on a 10% SDS-polyacrylamide gel, and then transferred onto a nitrocellulose membrane. Ponceau S staining of the membrane showed one protein at about 25 kD (pink, black arrow). Right panel - Western blot of SPLUNC1 using the Odyssey Imaging System verified that the 25 kD protein band shown in the left panel was SPLUNC1 (green band). **(B) **A representative fragmentation spectrum of peptide LYVTIPLGIK (amino acids 129 to 138) from in-gel trypsin-digested recombinant hSPLUNC1 protein after matching algorithm using SpectrumMill. The y-axis indicates the relative intensity of the fragment ions, where 100% is the total ion intensity of the spectrum. **(C) **Recombinant human SPLUNC1 protein markedly reduced *Mycoplasma pneumoniae *(Mp) growth in 96-well culture plates for 2 hrs. CFUs = Colony forming units. Data are expressed as means ± SEM (N = 5 replicates).

Our recent publication demonstrated that recombinant mouse SPLUNC1 protein significantly inhibited the growth of Mp in a dose-dependent manner [7]. To verify if human SPLUNC1 protein exerts a similar activity to the mouse counterpart in inhibiting Mp growth, we generated recombinant human SPLUNC1 (hSPLUNC1) protein using a baculovirus expression system. The purity and specificity of hSPLUNC1 protein were verified by Western blot and mass spectrometry (Figure 6A and 6B). As shown in Figure 6C, hSPLUNC1 inhibited Mp growth in a dose-dependent manner.

## Discussion

Our study has provided the evidence, for the first time, that SPLUNC1 regulation is in part under the control of TLR2 signaling. First, we demonstrated that Mp infection and TLR2 agonist treatment increased SPLUNC1 production in wild type mouse tracheal epithelial cells, and such an increase of SPLUNC1 was abrogated in TLR2^-/- ^tracheal epithelial cells. Second, we found that in human bronchial epithelial cell air-liquid interface cultures, Mp and TLR2 agonist also up-regulated SPLUNC1 production. Knockdown of TLR2 gene expression in human bronchial epithelial cells significantly attenuated SPLUNC1 induction following Mp infection or TLR2 agonist stimulation.

SPLUNC1 protein is an abundantly expressed and secreted protein in large airway epithelial cells. Elucidation of function and regulation of such an abundant protein is critical to understand the role of SPLUNC1 in airway homeostasis and disease processes. In the current study, we extended our previous findings that human SPLUNC1 protein, like its mouse counterpart, exerted inhibitory effects on Mp growth, further confirming its host defense function.

Regulation of abundant proteins in the lung including SPLUNC1 remains an active area of research. For example, surfactant proteins A and D, mainly produced by type II alveolar epithelial cells and Clara cells, are also abundant in airway lining fluid. They play a pivotal role in host defense against various bacterial infections. Although certain cytokines (e.g., IL-1) and bacterial infections are known to stimulate SP-A or SP-D production [32,33], the direct role of TLR signaling in surfactant protein regulation is poorly understood. Instead, previous studies suggest that SP-A dampens TLR2 signaling [34]. In our previous studies [7], we found that SPLUNC1 is also able to suppress TLR2 agonist (i.e., Pam3CSK4)-induced IL-8 production in NCI-H292 cells. Our current study demonstrates that TLR2 signaling is directly involved in SPLUNC1 up-regulation. Collectively, our data suggest that TLR2 activation leads to SPLUNC1 up-regulation, which in turn may dampen TLR2 signaling, leading to airway homeostasis following an infection or exposure to environmental stimuli. Our findings have important implications in clinical settings. For example, patients with dampened TLR2 signaling in the airways may not be able to generate sufficient amount of SPLUNC1 in response to an infection, and fail to eliminate the invading pathogen and resolve excessive inflammatory response. Indeed, we have reported that in allergic airways or under a Th2 cytokine milieu, lung or airway epithelial Mp clearance was impaired with reduced TLR2 expression [35]. Thus, any treatment aimed at appropriately enhancing TLR2 signaling in the airways has the great potential to restore the host defense function attributed to SPLUNC1.

The functional consequences of bacterial (e.g., Mp) infection-induced SPLUNC1 in airway mucosa need to be robustly studied in future experiments as SPLUNC1 may have multiple functions. For example, a recent study suggests that SPLUNC1 regulates airway surface liquid volume by protecting epithelial Na(+) channel (ENaC) from proteolytic cleavage [36]. Our current study did not address the impact of Mp-induced SPLUNC1 production on apical volume, ion transport or ENaC activity, but these additional experiments will be considered to advance our understanding of bacteria-induced SPLUNC1 production. We also realize that SPLUNC1 induction following Mp infection serves as one of the innate defense mechanisms utilized by airway epithelial cells to fight against the invading pathogens as other antimicrobial substances such as lactotransferrin can also be induced following Mp infection (unpublished data from the authors' group).

To define the mechanisms by which TLR2 signaling regulates SPLUNC1 production, we focused on the role of NF-κB because we previously reported that Mp infection not only increased TLR2 expression, but also activated NF-κB [17]. First, we found that an NF-κB inhibitor suppressed Mp-induced SPLUNC1 production in human bronchial epithelial cells. Second, conditional (doxycycline-induced) NF-κB activation in mouse tracheal epithelial cells was sufficient to increase SPLUNC1 expression. Therefore, our results suggest the involvement of NF-κB pathway in SPLUNC1 up-regulation. However, to further define how NF-κB regulates SPLUNC1 at the transcriptional level, more research approaches are needed, including chromatin immunoprecipitation (CHIP), electrophoretic mobility shift assay (EMSA) and promoter assays using various SPLUNC1 promoter deletion mutants. We realize that NF-κB pathway may not be the sole signaling pathway responsible for Mp- or TLR2 agonist-induced SPLUNC1 production. Future work is warranted to better understand regulation of SPLUNC1 under other transcription factors. For example, our preliminary data suggest that transcription factor heat shock factor-1 may also contribute to Mp-induced SPLUNC1 production.

We are aware of several limitations in the current study. First, SPLUNC1 regulation was not investigated in the context of other strains of bacteria that also utilize TLR2 signaling. For example, nontypeable *Haemophilus influenzae *(NTHi) and *Moraxella catarrhalis *(Mc) have been found in the airways of chronic lung diseases such as chronic obstructive pulmonary disease (COPD) [37,38]. Both NTHi and Mc have been reported to utilize TLR2 signaling to induce inflammatory cytokine responses [39,40]. These additional strains of bacteria will be included in our future studies. Second, the in vivo role of airway epithelial TLR2 signaling in SPLUNC1 production was not addressed in the current study. This can be done by overexpressing TLR2 exclusively in airway epithelial cells of TLR2^-/- ^mice that will be infected with Mp or treated with a TLR2 agonist. Third, Bingle and co-workers performed co-localization study of SPLUNC1 with mucin MUC5AC in human airway tissues, and clearly demonstrated that goblet cells in human airways do not express SPLUNC1 [16]. In the current study, we did not focus on identifying the cellular sources of SPLUNC1. However, in our preliminary co-localization study of SPLUNC1 and Clara cell secretory protein (CCSP or CC10) in air-liquid interface cultured wild-type C57BL/6 mouse tracheal epithelial cells, some CCSP (+) cells were found to co-express SPLUNC1 protein. Future studies are needed to clarify the impact of TLR2 and NF-κB on epithelial phenotypes (e.g., Clara cells and ciliated epithelial cells) and associated regulation of SPLUNC1 expression. Lastly, SPLUNC1 secretion was measured without normalization to the cell numbers under various cell culture conditions. Future studies will be performed to normalize SPLUNC1 secretion by cell numbers to avoid the potential impact of Mp or Pam3CSK4 on cell proliferation.

## Conclusions

In summary, airway SPLUNC1 production is up-regulated following bacterial (i.e., Mp) infection and TLR2 agonist stimulation. Understanding the regulation of SPLUNC1 by TLR2 signaling will help design novel therapeutic approaches to restore SPLUNC1 levels in hosts with allergic diseases and cigarette smoke-associated diseases (e.g., COPD) which exhibit impaired SPLUNC1 production [13,41].

## List of Abbreviations

ALI: Air-liquid interface; CC10-CA-IKKβ: Constitutively active version of inhibitor of κB (IκB) kinase-beta under transcriptional control of the rat CC10 promoter; Mp: *Mycoplasma pneumoniae*; NF-κB: Nuclear factor kappa B; NHBE: Normal human bronchial epithelial cells; shRNA: Short hairpin RNA; SPLUNC1: Short palate, lung, and nasal epithelium clone 1; TLR2: Toll-like receptor 2.

## Competing interests

The authors declare that they have no competing interests.

## Authors' contributions

HWC, FG, and JT designed the experiments. HWC, YMJ-H, and RJM wrote the manuscript. HWC, FG, JT, QW, GZ, NR, SC, MM, SS, DJ, NM, and GCS performed the epithelial cell cultures and recombinant SPLUNC1 protein experiments. All authors read and approved the final manuscript.
